# Effects of levodopa on gut bacterial antibiotic resistance in Parkinson’s disease rat

**DOI:** 10.3389/fnagi.2023.1122712

**Published:** 2023-02-07

**Authors:** Shuo Sheng, Xianwei Li, Shuo Zhao, Changqing Zheng, Feng Zhang

**Affiliations:** ^1^Key Laboratory of Basic Pharmacology of the Ministry of Education, Joint International Research Laboratory of Ethnomedicine of the Ministry of Education and Key Laboratory of Basic Pharmacology of Guizhou Province, Zunyi Medical University, Zunyi, Guizhou, China; ^2^Electron Microscopy Room of School of Basic Medicine, Zunyi Medical University, Zunyi, Guizhou, China; ^3^Laboratory Animal Center, Zunyi Medical University, Zunyi, Guizhou, China; ^4^The Collaborative Innovation Center of Tissue Damage Repair and Regeneration Medicine of Zunyi Medical University, Zunyi, Guizhou, China

**Keywords:** Parkinson’s disease, levodopa, gut microbiota, antibiotic, resistance gene

## Abstract

The second most prevalent neurodegenerative ailment, Parkinson’s disease (PD), is characterized by both motor and non-motor symptoms. Levodopa is the backbone of treatment for PD at the moment. However, levodopa-induced side effects, such as dyskinesia, are commonly seen in PD patients. Recently, several antibiotics were found to present neuroprotective properties against neurodegenerative and neuro-inflammatory processes, which might be developed to effective therapies against PD. In this study, we aimed to identify if levodopa treatment could influence the gut bacterial antibiotic resistance in PD rat. Fecal samples were collected from healthy rats and 6-OHDA induced PD rats treated with different doses of levodopa, metagenomic sequencing data showed that levodopa resulted in gut bacteria composition change, the biomarkers of gut bacteria analyzed by LEfSe changed as well. More interestingly, compared with levodopa (5 mg/kg)-treated or no levodopa-treated PD rats, levodopa (10 mg/kg) caused a significant decrease in the abundance of tetW and vanTG genes in intestinal bacteria, which were related to tetracycline and vancomycin resistance, while the abundance of AAC6-lb-Suzhou gene increased apparently, which was related to aminoglycosides resistance, even though the total quantity of Antibiotic Resistance Gene (ARG) and Antibiotic Resistance Ontology (ARO) among all groups did not significantly differ. Consequently, our results imply that the combination of levodopa and antibiotics, such as tetracycline and vancomycin, in the treatment of PD may decrease the amount of corresponding antibiotic resistance genes in gut bacteria, which would give a theoretical basis for treating PD with levodopa combined with tetracycline and vancomycin in the future.

## Introduction

Parkinson’s disease (PD) is a degenerative neurologic illness that worsens over time. This disease is predicted to increase in prevalence as the aging of the population. As a result of the clinical presentation, which includes both motor and non-motor symptoms such bradykinesia, rest tremor, rigidity, and hunched posture, the movement condition progresses and significantly lowers quality of life (Hayes, 2019). According to histopathology, Parkinson’s disease (PD) is characterized by the buildup of neuronal Lewy bodies and the specific death of dopamine (DA) neurons in the substantia nigra pars compacta (SNpc) (Lotankar et al., 2017). DA neuronal loss might result from apoptosis and autophagy (Anglade et al., 1997). The presynaptic protein α-synuclein, which is neuropathologically and genetically connected to Parkinson’s disease (PD), α-synuclein aggregation is a sequential process that results in the accumulation of inert fibrils and oligomeric species inside of neurons, the SNpc dopaminergic neurons appear to be especially susceptible to the effects of α-synuclein aggregates (Song et al., 2015; Sampson et al., 2016; Miki et al., 2017). However, the primary cause of PD remains unclear.

For its impact on disability and cost-effectiveness, levodopa taken orally is without a doubt the “gold standard” of PD therapies. Levodopa medication has been shown to improve Parkinson’s symptoms in patients, most likely due to the striatum’s conversion of DA precursor to DA (LeWitt, 2015). Nonetheless, as the disease progresses and disease duration increases, symptoms associated with PD do not respond well to levodopa treatment, or appear to be resistant to levodopa (Vorovenci et al., 2016). This so-called levodopa resistance could make clinicians and researchers confused, as patients often have levodopa-responsive dystonia accompanied by levodopa-resistant symptoms and signs (Nonnekes et al., 2016). For PD patients who cannot be controlled by drugs alone, surgical methods, such as unilateral pallidotomy and deep brain stimulation, have positive outcomes (Radhakrishnan and Goyal, 2018).

A potentially ground-breaking method for treating Parkinson’s disease has evolved that uses antibiotics to their advantage. Numerous experimental and pre-clinical studies have shown that antibiotics may be used as neuroprotective drugs. Additionally, evidence suggests that antibiotics are helpful in reducing neuro-inflammation and mitochondrial dysfunction in addition to avoiding the production of α-synuclein (Yadav et al., 2021). In PD animal models, tetracyclines conferred DA neuroprotection *via* its anti-inflammatory, anti-oxidant and anti-apoptotic activities, doxycycline can reduce the production of mitochondrial-derived reactive oxygen species and prevent the aggregation and seeding of recombinant α-synuclein, rats exposed to rotenone-induced motor deficits can be improved by minocycline, which increased TH expression and inhibited the release of ROS and NO in the SN (Sun et al., 2019; Dominguez-Meijide et al., 2021; Rahmani et al., 2022). Recently, another MPTP-induced PD mice model study showed that, in pole and traction tests, mice with motor dysfunction significantly improved after receiving vancomycin as a pretreatment, which could inhibit the metabolism of DA, and vancomycin pretreatment also can alleviate neuroinflammation and decrease the expression of TLR4/MyD88/NF-κB/TNF-α signaling pathway in both brain and gut through reducing the number of astrocytes and microglial cells in the (SNpc) (Cui et al., 2022). However, on the other hand, the use of antibiotics caused antibiotic resistance increased in bacterial pathogens, which could make antibiotics ineffective in treating bacterial infections in the body. The gut microbiota is thought to represent a reservoir for the potential transmission of antibiotic resistance genes from commensals to pathogens, also known as the gut resistome (Montassier et al., 2021). Thus, this study examined the effects of different doses of levodopa on intestinal microflora structure and antibiotic resistance genes in 6-OHDA-induced PD rats. Particularly, these results would provide a theoretical basis for the future use of levodopa combined with antibiotics in the treatment of PD.

## Results

### Levodopa treatment changed gut bacteria composition.

**Figure 1:**
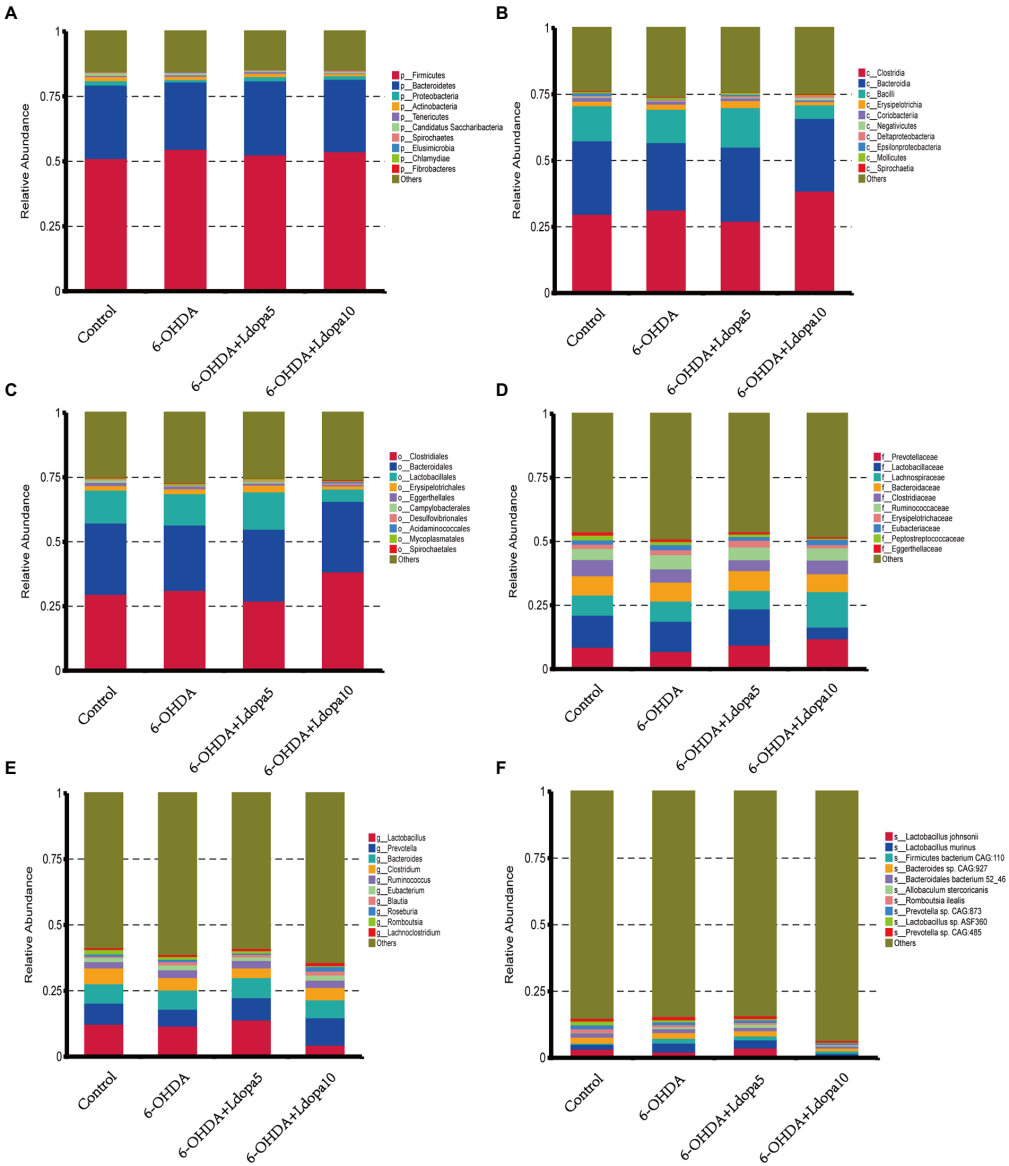
Gut bacteria composition of the 10 most abundant taxa was exhibited. Histogram of relative abundance in 6-OHDA, 6-OHDA+Ldopa5, 6-OHDA+Ldopa10 and control groups at phylum **(A)**, class **(B)**, order **(C)**, family **(D)**, genus **(E)** and species **(F)** level was discerned. The horizontal axis indicates the sample name, the vertical axis shows the relative proportion of annotations to a particular type of taxa, the taxa category corresponding to each color block are shown on the right side of the bar chart.

**Figure 2:**
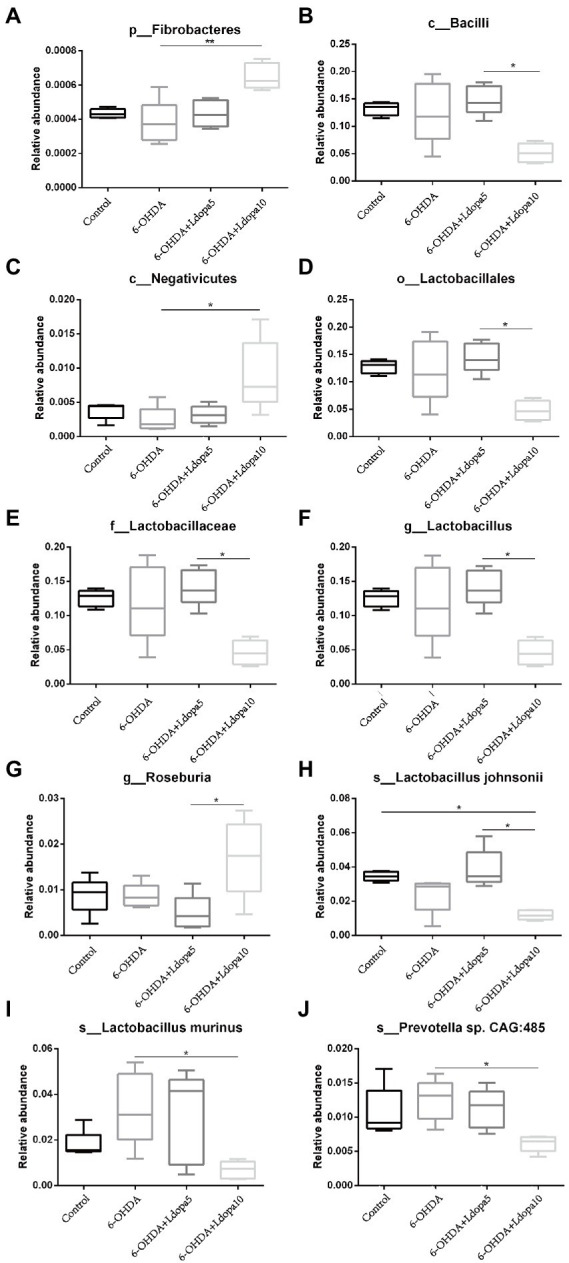
Differences of bacterial taxa of the 10 most abundant taxa were shown. The relative abundance of differentially abundant bacteria taxa in gut microbiota among control, 6-OHDA and levodopa-treated 6-OHDA groups at phylum level **(A)**, class level **(B,C)**, order level **(D)**, family level **(E)**, genus level **(F,G)** and species level **(H–J)** was detected. The horizontal axis is sample grouping, the vertical axis is the relative abundance of the corresponding taxa, the horizontal line represents the two groups with a significant difference, and none means there is no difference between the two groups. The asterisks indicated statistical significance tested using one-way ANOVA followed by Dunnett’s test (*p < 0.05, **p < 0.01).

Based on the relative abundance table of different classification levels, the composition and relative abundance of gut bacteria in 6-OHDA-induced PD rat (6-OHDA), 6-OHDA-induced PD rat treated with 5 mg/kg levodopa (6-OHDA+Ldopa5), 6-OHDA-induced PD rat treated with 10 mg/kg levodopa (6-OHDA+Ldopa10) and control groups were examined focusing on the 10 most abundant taxa at phylum-, class-, order-, family-, genus-and species-levels, respectively, and the rest taxa were set as others. The relative abundance histogram of the corresponding taxa annotation results of each group was drawn at different classification levels (Figure 1). In detail, at the phylum level, the relative abundance of *Fibrobacteres* increased apparently in 6-OHDA+Ldopa10 group compared to 6-OHDA group (Figure 2A). At the class level, the relative abundance of *Bacilli* decreased obviously in 6-OHDA+Ldopa10 group compared to 6-OHDA+Ldopa5 group (Figure 2B), and the relative abundance of *Negativicutes* increased strikingly in 6-OHDA+Ldopa10 group compared to 6-OHDA group (Figure 2C). At the order level, the relative abundance of *Lactobacillales* decreased notably in 6-OHDA+Ldopa10 group compared to 6-OHDA+Ldopa5 group (Figure 2D). At the family level, the relative abundance of *Lactobacillaceae* decreased prominently in 6-OHDA+Ldopa10 group compared to 6-OHDA+Ldopa5 group (Figure 2E). At the genus level, the relative abundance of *Lactobacillus* decreased remarkably in 6-OHDA+Ldopa10 group compared to 6-OHDA+Ldopa5 group (Figure 2F), and the relative abundance of *Roseburia* increased prominently in 6-OHDA+Ldopa10 group compared to the 6-OHDA+Ldopa5 group (Figure 2G). At the species level, the relative abundance of *Lactobacillus johnsonii* decreased significantly in 6-OHDA+Ldopa10 group compared to 6-OHDA and 6-OHDA+Ldopa5 groups (Figure 2H), while the relative abundance of *Lactobacillus murinus* and *Prevotella* sp. *CAG:485* decreased distinctly in 6-OHDA+Ldopa10 group compared to 6-OHDA group (Figures 2I,J). These results indicated that an increase in dosage of levodopa could potentially change the gut bacteria composition in 6-OHDA-induced PD rats.

### Levodopa treatment changed biomarkers of gut bacteria

**Figure 3:**
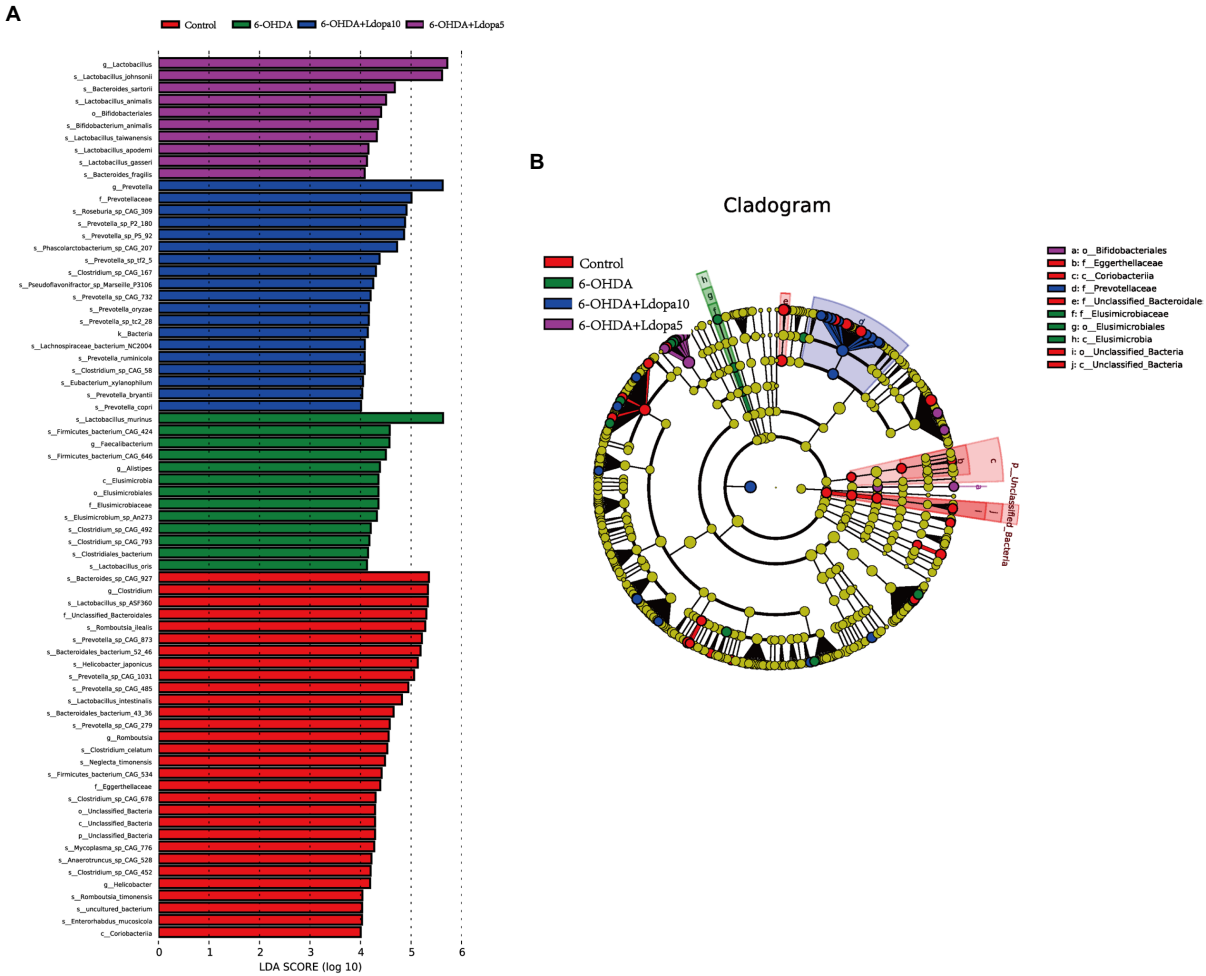
LEfSe comparison of gut microbiome was performed. Only taxa meeting an LDA significance threshold >4 were shown in the figures, namely, the biomarker with statistical difference among groups. **(A)** LDA scores of gut microbiota for the 6-OHDA, 6-OHDA+Ldopa5, 6-OHDA+Ldopa10 and control groups, the length of the bar chart represents the LDA Score. **(B)** Evolutionary branching diagram of the LEfSe results for the 6-OHDA, 6-OHDA+Ldopa5, 6-OHDA+Ldopa10 and control groups, circles radiating from inside out represent taxonomic levels from phylum to species, each small circle at a different classification level represents a classification at that level, and the size of the small circle diameter is proportional to the size of the relative abundance. The taxa with no significant difference were uniformly colored yellow, and the biomarkers were colored with the following groups, the taxa names represented by letters are shown on the right of the diagram.

Next, LEfSe was performed to analyze the differentially abundant of gut bacteria among the four groups. In order to screen the species biomarkers with significant differences between groups, the rank sum test was used to detect the different species in different groups, and linear discriminant analysis (LDA) was used to achieve dimension reduction and assess the impact of the different species, namely, LDA score. LEfSe analysis results of different species between groups included histogram of LDA value distribution and evolutionary clade diagram (phylogenetic distribution), which showed that 75 bacterial communities were different among control, 6-OHDA, 6-OHDA+Ldopa5 and 6-OHDA+Ldopa10 groups. There were 30 biomarkers in control, 13 biomarkers in 6-OHDA, 10 biomarkers in 6-OHDA+Ldopa5 and 19 biomarkers in 6-OHDA+Ldopa10 (Figure 3A). The biomarkers in control group mainly included *Bacteroides_sp_CAG_927* (species), *Clostridium* (genus), *Lactobacillus_sp_*ASF360 (species), *Unclassified_Bacteroidales* (family), and *Romboutsia_ilealis* (species), among which the most abundant was *Bacteroides_sp_CAG_927* (species). The biomarkers in 6-OHDA group mainly included *Lactobacillus_murinus* (species), *Firmicutes_bacterium_CAG_424* (species), *Faecalibacterium* (genus), *Firmicutes_bacterium_CAG_646* (species), and *Alistipes* (genus), among which the most abundant was *Lactobacillus_murinus* (species). The biomarkers in 6-OHDA+Ldopa10 group mainly included *Prevotella* (genus), *Prevotellaceae* (family), *Roseburia_sp_CAG_309* (species), *Prevotella _sp_P2_180* (species), and *Prevotella _sp_P5_92* (species), in which the most abundant was *Prevotella* (genus). The biomarkers in 6-OHDA+Ldopa5 group mainly included *Lactobacillus* (genus), *Lactobacillus_johnsonii* (species), *Bacteroides_sartorii* (species), *Lactobacillus_animalis* (species), and *Bifidobacteriates* (order), in which the most abundant was *Lactobacillus* (genus). The cladogram showed significant changes in each group (Figure 3B).

### Levodopa treatment changed the ARO composition

**Figure 4:**
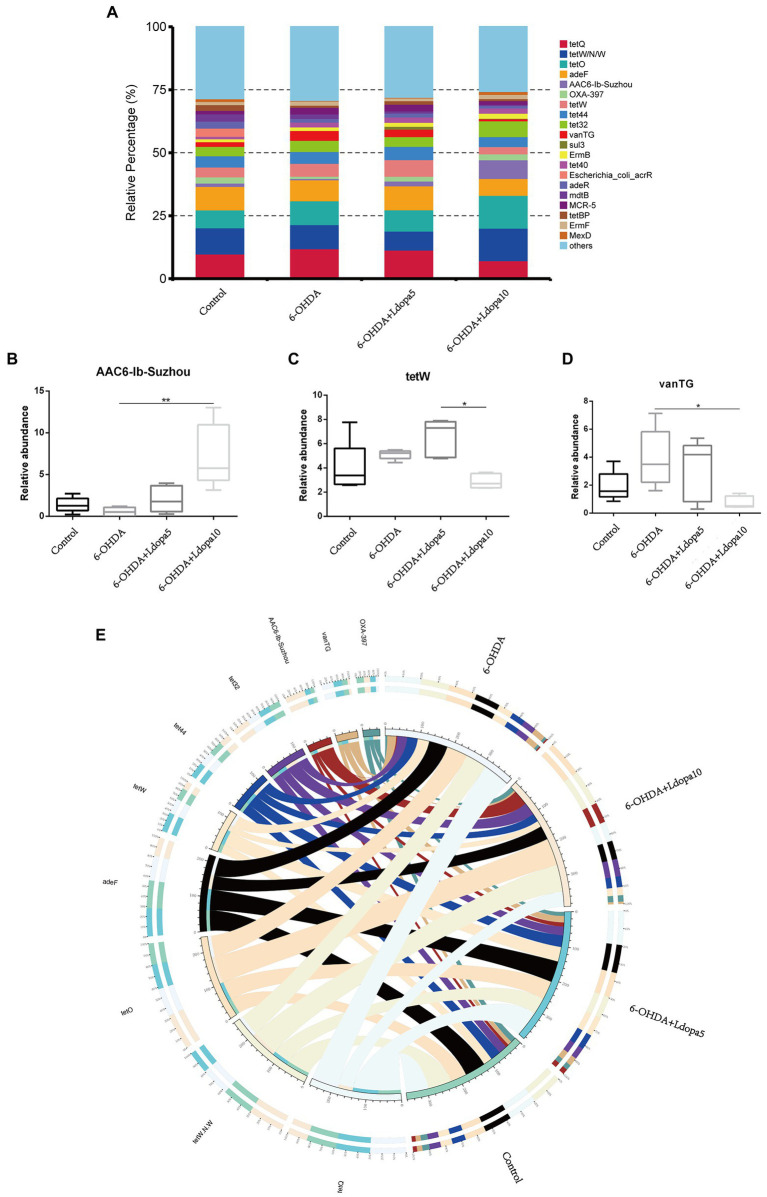
Relative abundances of the top 20 ARO were analyzed. **(A)** The relative abundance of top 20 ARO in control, 6-OHDA, 6-OHDA+Ldopa5 and 6-OHDA+Ldopa10 groups, the relative abundance of others is the sum of the relative abundance of non-top 20 ARO. **(B–D)** The relative abundance of differentially ARO in gut microbiota between 6-OHDA group and levodopa-treated 6-OHDA groups was discerned. The asterisks indicated statistical significance tested using one-way ANOVA followed by Dunnett’s test (*p < 0.05, **p < 0.01). **(E)** The circle diagram was divided into two parts, the right is the group information and the left is the ARO information. Different colors of inner circle represent different groups and ARO, scale is the relative abundance, the left side of the inner circle is the sum of the relative abundance of each group of an ARO, and the right is the sum of the relative abundance of each ARO in a group. The left side of the outer circle is the relative percentage content of each group in an ARO, and the right side of the outer circle was the relative percentage content of each ARO in a group.

Resistance genes are ubiquitous in both human gut microbes and other environmental microbes. Comprehensive Antibiotic Resistance Database (CARD) is a newly emerged resistance gene database in recent years. The core component of CARD is ARO, which integrates the antibiotic sequence, antibiotic resistance and function mechanism, et al. Using the Resistance Gene Identifier program, the unigenes were aligned to the CARD. Based on the relative abundance of resistance gene, the common top 20 AROs identified in 6-OHDA, 6-OHDA+Ldopa5, 6-OHDA+Ldopa10 and control groups were tetQ, tetW/N/W, tetO, adeF, AAC6-Ib-Suzhou, OXA-397, tetW, tet44, tet32, vanTG, sul3, ErmB, tet40, *Escherichia_coli*_acrR, adeR, mdtB, MCR-5, tetBP, ErmF and MexD (Figure 4A). In comparison to the 6-OHDA group, the relative percentage of AAC6-Ib-Suzhou in the 6-OHDA+Ldopa10 group was greater (Figure 4B). In comparison to the 6-OHDA+Ldopa5 group, the relative percentage of tetW in the 6-OHDA+Ldopa10 group was lower (Figure 4C). In comparison to the 6-OHDA group, the relative percentage of vanTG in the 6-OHDA+Ldopa10 group was lower (Figure 4D). To more intuitively observe the proportion of ARO abundance in each group on the whole and more intuitively display the overall distribution of ARO abundance, the AROs with the top10 abundance was selected to draw the overview circle diagram (Figure 4E).

### The total number of ARG and ARO did not alter significantly after levodopa treatment

**Figure 5:**
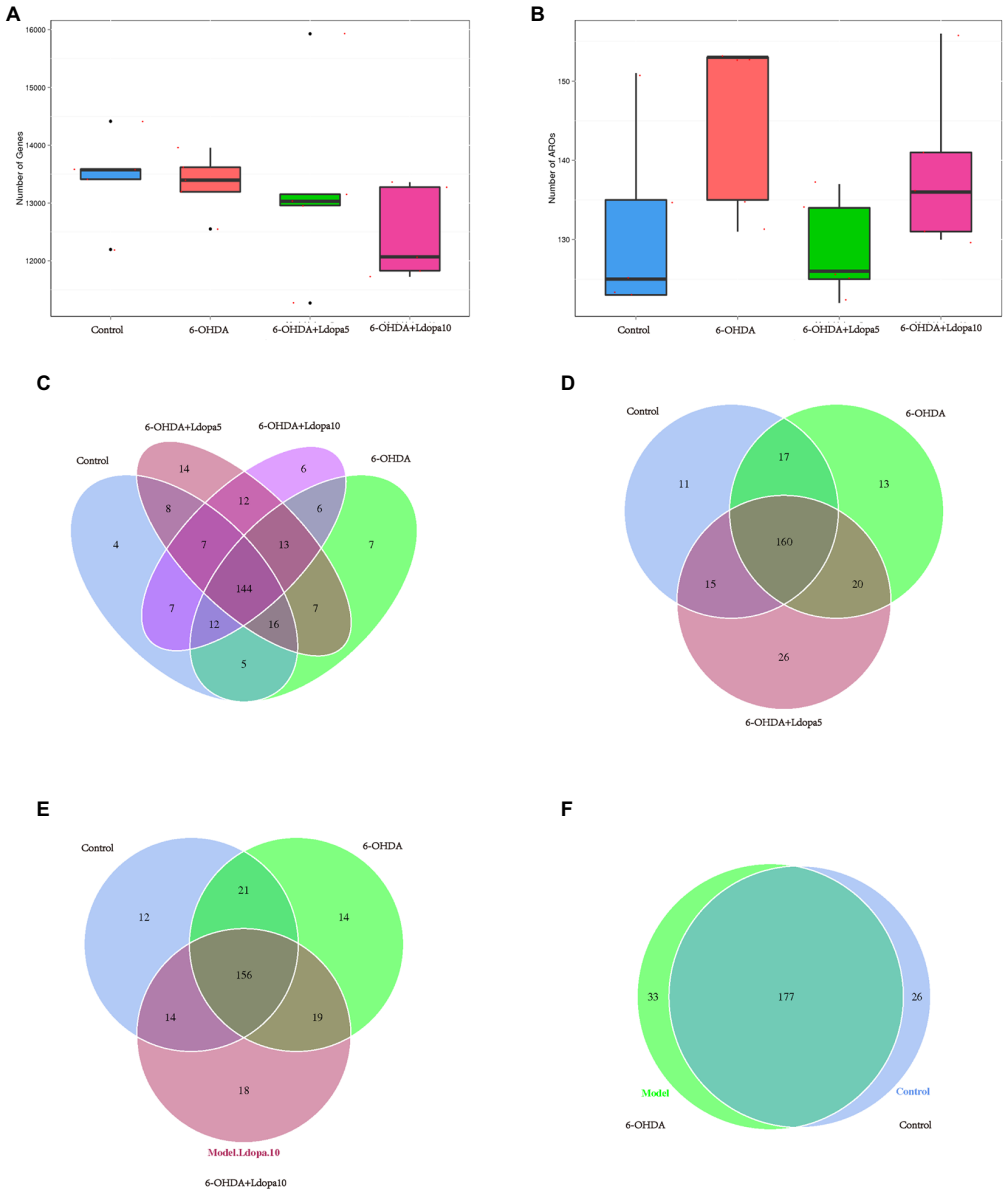
Antibiotic resistance genes shared among the four groups. **(A)** Number of antibiotic resistance genes in control, 6-OHDA, 6-OHDA+Ldopa5 and 6-OHDA+Ldopa10 groups is shown. **(B)** Number of ARO in control, 6-OHDA, 6-OHDA+Ldopa5 and 6-OHDA+Ldopa10 groups was indicated. **(C–F)** Each circle represents a group in the Venn diagram, the number of overlapped parts represent the number of resistance genes shared among groups, and the number without overlap represent the number of specific resistance genes in the group.

In order to investigate the differences in the number of ARG and ARO between the sample groups, the box maps of the number of ARG and ARO between the sample groups were drawn, which showed that the number of ARG and ARO among all groups did not significantly differ (Figures 5A,B). A Venn diagram was also used to examine how the four groups’ shared ARGs were composed. The Venn diagram for the ARG number classified by the four component approaches is depicted in Figure 5C. The four component methods complemented each other in classification. Four, 7, 14 and 6 ARGs were uniquely correctly classified by control, 6-OHDA, 6-OHDA+Ldopa5 and 6-OHDA+Ldopa10 groups, respectively. Moreover, the Venn diagram for the ARG number classified by the three component approaches is depicted in Figure 5D. The three component methods complemented each other in classification. Eleven, 13 and 26 ARGs were uniquely correctly classified by control, 6-OHDA and 6-OHDA+Ldopa5 groups, respectively. In addition, the Venn diagram for the ARG number classified by the three component methods. The three component methods complemented each other in classification. Twelve, 14 and 18 ARGs were uniquely correctly classified by control, 6-OHDA and 6-OHDA+Ldopa10 groups, respectively (Figure 5E). Furtherly, the Venn diagram for the ARG number classified by the two component methods. The two component methods complemented each other in classification. Thirty-three and 26 ARGs were uniquely correctly classified by control and 6-OHDA groups (Figure 5F).

## Discussion

In this study, levodopa resulted in different changes in intestinal flora at different species levels in 6-OHDA-induced PD rat model. In the 10 most abundant taxa analysis, we found apparent changes in the abundance of *Fibrobacteres* (phylum), *Negativicutes* (class), *Lactobacillus murinus* (species) and *Prevotella* sp. *CAG:485* (species) between 6-OHDA and 6-OHDA+Ldopa10 groups were shown. Furtherly, between 6-OHDA+Ldopa10 and 6-OHDA+Ldopa5 groups, the abundance of *Bacilli* (class), *Lactobacillales* (order), *Lactobacillaceae* (family), *Lactobacillus* (genus), *Roseburia* (genus) and *Lactobacillus johnsonii* (species) was discerned. However, the gut bacteria composition of the 10 most abundant taxa in 6-OHDA+Ldopa5 group did not change significantly compared to the 6-OHDA group. These findings suggested that the intestinal flora of PD-rats may respond differently to various levodopa dosages, and the change of intestinal flora induced by long-term high dose of levodopa might be one of the causes of its side effects.

LEfSe was employed to analyze the specific biomarkers of gut bacteria. This analysis revealed that *Prevotella* (genus), *Prevotellaceae* (family), *Roseburia_sp_CAG_309* (species), *Prevotella _sp_P2_180* (species), *Prevotella _sp_P5_92* (species), *Phascolarctobacterium_sp_CAG_207* (species), *Prevotella_sp_tf_2_5* (species), *Clostridium_sp_CAG_167* (species), *Pseudoflavonifractor_sp_Marseille_P3106* (species), *Prevotella_sp_CAG_732* (species), *Prevotella_oryzae* (species), *Prevotella_sp_tc2_28* (species), *Bacteria* (kingdom), *Lachnospiraceae_bacterium_NC2004* (species), *Prevotella_ruminicola* (species), *Clostridium_sp_CAG_58* (species), *Eubacterium_xylanophilum* (species), *Prevotella_bryantii* (species) and *Prevotella_copri* (species) were increased after 10 mg/kg levodopa treatment. *Lactobacillus* (genus), *Lactobacillus_johnsonii* (species), *Bacteroides_sartorii* (species), *Lactobacillus_animalis* (species), *Bifidobacteriates* (order), *Bifidobacterium_animalis* (species), *Lactobacillus_taiwanensis* (species), *Lactobacillus_apodemi* (species), *Lactobacillus gasseri* (species) and *Bacteroides_fragilis* (species) were increased after 5 mg/kg levodopa treatment. Among the biomarkers of levodopa-treated groups, previous studies identified that the genus *Prevotella* played major roles in balancing health and sickness (Tett et al., 2021), and there was increased acceptance of applying allochthonous probiotic *Lactobacillus* in fermented foods and supplements to maintain health and prevent disease (Heeney et al., 2018).

Numerous studies have demonstrated associations between various ARO and the significant antibiotic resistance of human microorganisms (Sommer et al., 2010; Almeida et al., 2020). The relative abundance of AAC6-Ib-Suzhou, tetW, and vanTG changed noticeably following 10 mg/kg levodopa treatment among the ARO found to be present in the gut microbiomes of the individuals in these four groups of this investigation. The acquisition of DNA encoding proteins that imparted resistance by ATP-dependent efflux, enzymatic inactivation of tetracycline, or ribosome protection was confirmed to be a common mechanism by which bacteria developed resistance to tetracycline by current evidence (Chopra and Roberts, 2001). To date, 60 different classes of tetracycline resistance gene had been well studied, including oxytetracycline resistance genes, which encode active efflux pumps, ribosomal protection proteins (RPPs) and inactivating enzymes, and tetW is one of the RPP genes (Warburton et al., 2016). The main cause of vancomycin resistance in Gram-positive bacteria is the substitution of the peptidoglycan precursor’s d-alanyl-d-Ala target with d-alanyl-d-lactate or d-Ala-d-Ser, to which vancomycin has a weak binding affinity, and vanTG is a resistance gene to vancomycin (Meziane-Cherif et al., 2015). A common strategy for developing resistance to aminoglycosides is the enzyme-mediated transfer of an acetyl group from acetyl-CoA to the 6′-amine group of the antibiotic molecule. The aminoglycoside 6’-N-acetyltransferase type lb. [AAC (6′)-lb] enzyme in integrons, transposons, plasmids, and chromosomes of Gram-negative bacteria, is responsible for the majority of aminoglycosides-resistant, and AAC6-Ib-Suzhou is a resistance gene to aminoglycosides (Reeves et al., 2020).

Although a number of pharmaceutical substances could reduce PD’s motor and non-motor symptoms by enhancing the nigrostriatal pathway’s performance, the disease’s progression cannot be stopped by these medications (Schapira, 2005). In fact, the DA precursor levodopa, the long-established gold standard in PD therapy, tries to treat PD motor symptoms by supplying the missing neurotransmitter. Levodopa’s treatment effectiveness does, however, have a tendency to wane over time and is accompanied by very unpleasant motor and/or psychological side effects for patients. Therefore, it is crucial that any future therapeutic research aim to delay DA neurodegeneration and preventing the progression of the disease (LeWitt, 2004; Schapira et al., 2006). In order to prevent the loss of DA neurons and reduce the progression of the disease, neuroprotection is anticipated to be the main focus of future therapeutic development methods for PD (Schapira, 1999), the quest for protective agents on PD is still ongoing (Palasz et al., 2020). Since they can reduce oxidative stress, protein misfolding, neuroinflammation, and mitochondrial dysfunction, antibiotics have attracted fresh investigation for their neuroprotective qualities.

Antibiotics enable critical therapies and are crucial for treating infectious diseases (Cook and Wright, 2022). Since their widespread use in human health began, antibiotics have profoundly altered the landscape of infectious diseases, allowing them to be brought under control and resulting in a sharp decline in the number of fatalities. Today, nevertheless, there is a resurgence of interest in antibiotics because of some unexpected side effects unrelated to their antibacterial activity. Therefore, it has been amply shown that sub-inhibitory concentrations of any antibiotic can alter the expression of a large number of gene transcripts in both prokaryotic and eukaryotic cells. Moreover, a number of antibiotics, particularly tetracyclines and-lactams, have impressive anti-inflammatory properties. Tetracyclines, have been shown to generate neuroprotection, limit the synthesis of pro-inflammatory molecules and the activity of matrix metalloproteinases, improve mitochondrial dysfunction and protein misfolding/aggregation, and reduce the activation of microglia. Minocycline and doxycycline, two frequently used semisynthetic second-generation tetracycline derivatives, shown potent neuroprotective efficacy in experimental neurodegenerative/neuropsychiatric illnesses with minimal toxicity, for instance, in a PD rat model, minocycline can prevent rotenone-induced neurotoxicity associated with upregulation of Nurr1 (Bortolanza et al., 2018; Sun et al., 2019). In traction and pole tests, a recent study showed that pretreatment with vancomycin reduced the motor impairment of PD mice. Vancomycin pretreatment did not affect the level of DA or the process of DA synthesis, but it did impede DA metabolism by downregulating striatal monoamine oxidase B expression (MAO-B). Furthermore, vancomycin pretreatment decreased the amounts of astrocytes and microglia in SNpc to diminish neuro-inflammation and downregulated the TLR4/MyD88/NF-B/TNF-signaling pathway in both the brain and intestine (Cui et al., 2022).

To conclude, this study revealed that levodopa therapy in PD rat model led to changes in resistance genes, which might be related to alterations in intestinal flora. Antibiotics have emerged as potential drugs to treat PD, but they still face great challenges, such as bacterial resistance (Yadav et al., 2021). Levodopa remains the drug of first choice for PD, although it generates several side effects (Lane, 2019; Cilia et al., 2020). According to findings of the present study, appropriate dose of levodopa could reduce the number of tetracycline and vancomycin resistance genes in intestinal bacteria. Therefore, the combination of these two antibiotics with levodopa might be more effective in PD treatment compared with levodopa alone. At the same time, to reduce the intestinal bacterial resistant gene thus decreased resistance gene transfer. However, our study is preliminary and was limited in power by the low number of rats, which need more experimental samples to be used in the further study. Moreover, as it is known that other organs may also be affected during treatment in a human condition, so using levodopa combined with antibiotics to treat PD rats and perform behavioral and pathological tests in further research will be more clinically relevant.

## Materials and methods

### Animals

We housed 6-week-old male SD rats with free access to food and water that weighed 180–200 g. The institutional Animal Care and Use Committee of Zunyi Medical University (Zunyi, China) gave its approval to all animal experiments.

### 6-OHDA lesion and levodopa treatment

The experiments were carried out as formerly reported (Zheng et al., 2021). In order to target the unilateral SNpc (coordinates AP-5.2 mm, ML-2.1 mm, DV-8.0 mm) relative to the bregma, rats were anesthetized and then immobilized in a stereotaxic frame, 6-OHDA solution was injected over 3 min with an infusion rate of 1 μl/min, followed by 3 min of equilibrium before retracting the needle. Prior to the experiment, all rats had recovered after 3 weeks. Then, for 12 weeks, a daily intraperitoneal injection of 5 mg/kg or 10 mg/kg levodopa was administered.

### Fecal DNA extraction

Following a 12-week course of levodopa therapy, feces were collected according to the previously reported methodology (Zhang and Wang, 2017), Each sample was briefly lysed using lysozyme-containing CTAB buffer. Chloroform, isoamyl alcohol (24:1), and isopropanol were used to extract the whole DNA. The precipitate was then twice rinsed with 75% ethanol before being dissolved in ddH_2_O, RNase A was then used to degrade the RNA.

### Metagenomic sequencing

For sample testing, on 1% agarose gels, the level of DNA degradation and potential contamination were observed, DNA concentration was measured using Qubit® dsDNA Assay Kit in Qubit® 2.0 Flurometer (Life Technologies, CA, United States), DNA concentrations greater than 1 ug are used to construct libraries with an OD value of 1.8 to 2.0. The input material for the DNA sample preparations was a total of 1 g of DNA per sample. Utilizing the NEBNext® Ultra™ DNA Library Prep Kit for Illumina (NEB, United States) in accordance with the manufacturer’s instructions, sequencing libraries were created, and index codes were added to assign sequences to specific samples. The DNA sample was broken up to 350 bp-sized fragments using sonication, after which it was end-polished, A-tailed, and ligated with the full-length adaptor for Illumina sequencing and additional PCR amplification. Finally, PCR products were purified using the AMPure XP system, and libraries underwent size distribution analysis with an Agilent 2,100 Bioanalyzer and real-time PCR quantification. On a cBot Cluster Generation System, the index-coded samples were clustered in accordance with the manufacturer’s recommendations. The library preparations were sequenced on an Illumina HiSeq platform after cluster generation, and paired-end reads were produced.

### Sequencing results pretreatment

To obtain the clean data needed for further analysis, Readfq (V8^1^) was used to preprocess the raw data collected from the Illumina HiSeq sequencing technology. These were the precise processing steps: reads were first removed if they contained low-quality bases (default quality threshold value ≤38) above a predetermined portion (default length of 40 bp); then, reads were removed if the N base reached a predetermined percentage (default length of 10 bp); and finally, reads were removed if they shared the overlap with the adapter above a predetermined length (default length of 15 bp). Given the potential for host contamination in samples, clean data had to be sent to the host database, and Bowtie2.2.4 software was used by default to filter out reads of host origin (Bowtie2.2.4^2^), the following were the parameters: –end-to-end, −-sensitive, -I 200, −X 400 (Karlsson et al., 2012, 2013).

### Metagenome assembly

The SOAPdenovo software (V2.04^3^) was used to assemble and analyze the clean data(Luo et al., 2012), the following were the parameters: -d 1, −M 3, −R, −u, -F, -K 55 (Scher et al., 2013; Qin et al., 2014; Brum et al., 2015; Feng et al., 2015). MEGAHIT software (v1.0.4-beta) could be used to assemble the Clean Data for samples taken from complex environments, such as water, soil, and so forth, and the parameters are-presets meta-large (−- min-count 2 --k-min 27 --k-max 87 --k-step 10). Then, the built Scaftigs were severed from N connection and the Scaftigs were left without N (Mende et al., 2012; Nielsen et al., 2014; Qin et al., 2014). All samples’ clean data were compared to each Scaffolds correspondingly using Bowtie2.2.4 software to acquire the PE reads not used and the parameters were: --end-to-end, −-sensitive, -I 200, −X 400 (Qin et al., 2014).

### Gene prediction and abundance analysis

The MetaGeneMark (V2.10) software, available at http://topaz.gatech.edu/GeneMark/, predicted all ORFs for the scaftigs (≥500 bp) assembled from single and mixed samples, and filtered with the length information shorter than 100 nt from the anticipated result *via* default parameters (Qin et al., 2010, 2014; Li et al., 2014; Nielsen et al., 2014; Zeller et al., 2014). For ORF predicted, CD-HIT software (V4.5.8^4^; Li and Godzik, 2006; Fu et al., 2012) was adopted to obtain the unique initial gene catalog [the genes here referred to the nucleotide sequences coded by unique and continuous genes (Sunagawa et al., 2015)]. The parameters option (Zeller et al., 2014; Sunagawa et al., 2015) were-c 0.95, −G 0, -aS 0.9, −g 1, −d 0. Using Bowtie2.2.4, the clean data from each sample was mapped to the initial gene catalog. The number of reads was changed to reflect the number of genes mapped in each sample using the parameter settings --end-to-end, −-sensitive, -I 200, −X 400 (Li et al., 2014; Qin et al., 2014). The gene which the number of reads ≤2 (Qin et al., 2012; Li et al., 2014) was filtered in each sample and the gene catalog (Unigenes) was eventually used for subsequent analysis. Based on the amount of mapped reads and the length of gene, statistic analysis on the abundance information of each gene in each sample was done. The format was as follow, “*r”* represented the number of reads mapped to the genes and “*L*” represented gene’s length (Qin et al., 2010; Karlsson et al., 2012; Cotillard et al., 2013; Le Chatelier et al., 2013; Zeller et al., 2014; Villar et al., 2015). The abundance of each gene in each sample in the gene catalog served as the basis for the fundamental data of statistics, core-pan gene analysis, correlation analysis of samples, and Venn diagram analysis of number of genes.

### Taxonomy prediction

The DIAMOND program (V0.9.9^5^; Buchfink et al., 2015) was used to blast the Unigenes to the sequences of Bacteria, Fungi, Archaea, and Viruses from the NCBI NR database (Version: 2018-01-02^6^) using the parameter-e 1e-5. The LCA algorithm was used for system classification of MEGAN (Huson et al., 2011) software to ensure the species annotation information of sequences. For the finally aligned results of each sequence, each sequence may have multiple aligned results, and the result of which the e value the smallest e value * 10 (Oh et al., 2014) was chosen. Based on the LCA annotation result and the gene abundance table, a table including the number of genes and the abundance information of each sample in each taxonomy hierarchy (kingdom, phylum, class, order, family, genus, and species) was created. The abundance of one specie in one sample was labeled as the sum of the gene abundances for the specie. A specie’s gene number in a sample was equal to the number of genes with nonzero abundance. Based on the abundance table of each taxonomic hierarchy, Krona analysis was used to display the generation situation of relative abundance, the abundance cluster heat map, PCA (Avershina et al., 2013; R ade4 package, Version 2.15.3), and NMDS (Noval Rivas et al., 2013; R vegan package, Version 2.15.3). Anosim analysis was used to examine the difference between groups (R vegan package, Version 2.15.3). To find the distinct species among groups, metastats and LEfSe analysis were utilized. In Metastats analysis, the permutation test across groups was performed to obtain *p* value for each taxonomy, and then the Benjamini and Hochberg False Discovery Rate was calculated to correct p value and obtain q value (White et al., 2009). The LEfSe program carried out the LEfSe analysis (the default LDA score is 3) (Segata et al., 2011). Finally, a random forest of 6-OHDA was created using the random forest (RandoForest) (Breiman, 2001; R pROC and randomForest packages, Version 2.15.3). By using MeanDecreaseAccuracy and MeanDecreaseGin, the relevant species were eliminated. Each group was then cross-validated (by default, 10 times), and the ROC curve was generated.

### Common functional database annotations

To blast Unigenes to a functioning database, DIAMOND software (V0.9.9) was used using the parameter setting of BLASTp, −e 1e-5 (Li et al., 2014; Feng et al., 2015). Functional databases were exempted from the study, including the KEGG (Kanehisa et al., 2006, 2014) database (Version 2018-01-01^7^), the eggnog (Powell et al., 2014) database (Version 4.5^8^), and the CAZy (Cantarel et al., 2009) database (Version 201,801^9^). The best Blast Hit for each sequence’s blast result was utilized for further investigation (Li et al., 2014; Bäckhed et al., 2015; Feng et al., 2015). A statistical study of the relative abundance of several functional hierarchies was annotated, with the relative abundance of each functional hierarchy being equal to the total of the relative abundances. The gene number table for each sample in each taxonomic hierarchy was obtained based on the function annotation result and gene abundance table. A function’s gene number in a sample matched the gene number that was ascribed to it, and the abundance was non-zero. The number of annotated genes was counted, the overall relative abundance situation was displayed, an abundance cluster heat map was displayed, and decrease-dimension PCA and NMDS analyzes were performed using the abundance tables of each taxonomic hierarchy. Anosim study of the difference between groups (inside) based on functional abundance, comparative analysis of metabolic pathways, and analyzes of functional difference between groups using Metastats and LEfSe were also carried out.

### Resistance gene annotation

With the parameter setting of BLASTp, evalue ≤1e-30, the Unigenes database^10^ (McArthur et al., 2013; Martínez et al., 2015; Jia et al., 2017) was aligned to the CARD database using the Resistance Gene Identifier (RGI) program. The aligned result was used to count the relative abundance of ARO. The abundance bar charts, abundance cluster heatmap, and the numerical difference between groups of resistance genes were all shown using the abundance of ARO. The distribution of resistance genes’ abundance in each sample, an analysis of the resistance genes’ species attribution, and a study of the resistance mechanisms of the resistance genes were also carried out.

### Statistical analysis

The data were shown as mean ± SEM. For the statistical comparison, one-way analysis of variance (ANOVA) was performed using SPSS statistical software. The Dunnett’s test was applied to all pairwise comparisons of means where analysis of variance revealed significant differences. Statistical significance was defined as *p* < 0.05.

## Data availability statement

The datasets presented in this study can be found in online repositories. The names of the repository/repositories and accession number(s) can be found below: https://www.ncbi.nlm.nih.gov/, PRJNA883557.

## Author contributions

SS and FZ designed and conceived the experiments, wrote and revised the manuscript. SS, SZ, CZ, and XL participated in the experiment performance. All authors read and approved by the final manuscript.

## Funding

This work was supported by special grant of academic new seedling cultivation and innovation exploration from Guizhou Science and Technology Department [Qian Ke He Ping Tai Ren Cai (2018)5772–036 and (2020)-012], Science and Technology Project of Zunyi City [Zun Shi Ke He HZ Zi (2021)No. 286 and (2022) No. 372], Natural Science Foundation of Guizhou Province [Qian Ke He Ji Chu – ZK (2022) No. 604], National Natural Science Foundation of China (No. 82160690), Science and Technology Foundation of Guizhou Province [No. ZK (2021)-014], Collaborative Innovation Center of Chinese Ministry of Education (No. 2020–39), Scientific Research Youth Project of Higher Education Department of Guizhou Province [Qian Jiao Ji (2022) No. 236] and a scholarship granted by the China Scholarship Council (No. 202108520054).

## Conflict of interest

The authors declare that the research was conducted in the absence of any commercial or financial relationships that could be construed as a potential conflict of interest.

## Publisher’s note

All claims expressed in this article are solely those of the authors and do not necessarily represent those of their affiliated organizations, or those of the publisher, the editors and the reviewers. Any product that may be evaluated in this article, or claim that may be made by its manufacturer, is not guaranteed or endorsed by the publisher.

## Footnotes

^1^ https://github.com/cjfields/readfq

^2^http://bowtiebio. sourceforge.net/bowtie2/index.shtml

^3^ http://soap.genomics.org.cn/soapdenovo.html

^4^ http://www.bioinformatics.org/cd-hit

^5^ https://github.com/bbuchfink/diamond/

^6^ https://www.ncbi.nlm.nih.gov/

^7^ http://www.kegg.jp/kegg/

^8^ http://eggnogdb.embl.de/#/app/home

^9^ http://www.cazy.org/

^10^ https://card.mcmaster.ca/
